# Health technology assessment in musculoskeletal radiology: the case study of EOSedge™

**DOI:** 10.1007/s11547-024-01832-9

**Published:** 2024-06-10

**Authors:** Rossella Tomaiuolo, Giuseppe Banfi, Carmelo Messina, Domenico Albano, Salvatore Gitto, Luca Maria Sconfienza

**Affiliations:** 1 Vita-Salute San Raffaele University, Milan, Italy; 2 IRCCS Istituto Ortopedico Galeazzi, Via Cristina Belgioioso 173, 20157 Milan, Italy; 3 Department of Biomedical Sciences for Health, University of Milan, Milan, Italy; 4 Department of Biomedical, Surgical, and Dental Sciences, University of Milan, Milan, Italy

**Keywords:** HTA (health technology assessment), EOSedge, EOS system, Radiography, Musculoskeletal

## Abstract

**Objectives:**

Health technology assessment (HTA) is a systematic process used to evaluate the properties and effects of healthcare technologies within their intended use context. This paper describes the adoption of HTA process to assess the adoption of the EOSedge™ system in clinical practice.

**Methods:**

The EOSedge™ system is a digital radiography system that delivers whole-body, high-quality 2D/3D biplanar images covering the complete set of musculoskeletal and orthopedic exams. Full HTA model was chosen using the EUnetHTA Core Model^®^ version 3.0. The HTA Core Model organizes the information into nine domains. Information was researched and obtained by consulting the manufacturers’ user manuals, scientific literature, and institutional sites for regulatory aspects.

**Results:**

All nine domains of the EUnetHTA Core Model^®^ helped conduct the HTA of the EOSedge, including (1) description and technical characteristics of the technology; (2) health problem and current clinical practice; (3) safety; (4) clinical effectiveness; (5) organizational aspects; (6) economic evaluation; (7) impact on the patient; (8) ethical aspects; and (9) legal aspects.

**Conclusions:**

EOS technologies may be a viable alternative to conventional radiographs. EOSedge has the same intended use and similar indications for use, technological characteristics, and operation principles as the EOS System and provides significant dose reduction factors for whole spine imaging compared to the EOS System without compromising image quality. Regarding the impact of EOS imaging on patient outcomes, most studies aim to establish technical ability without evaluating their ability to improve patient outcomes; thus, more studies on this aspect are warranted.

## Introduction

The development of healthcare technologies is steadily increasing, and the decision-makers must evaluate whether new technology should be used to replace the existing systems to maximize patients’ health under budget constraints. Health technology assessment (HTA) structures the complexity of the decision-making process, as it is a systematic process used to evaluate the properties and effects of healthcare technologies within the context of their intended use [1]. Therefore, HTA is a valid management tool for supporting decision-makers in adopting the most appropriate healthcare technologies (including pharmaceuticals, medical devices, in vitro diagnostic systems and other technology-based tools for disease prevention, diagnosis, or treatment) and also represents a non-negligible achievement in the definition of European Union health policies [2].

Health technology assessment of diagnostic technologies (laboratory tests and diagnostic imaging) has proven a handy tool, especially in well-defined diagnostic contexts [3, 4]. As the diagnostic phase is typically an intermediary step driving the medical decision [5], it is mandatory to establish the therapeutic impact on the patient’s outcome and not just the technical performance of the diagnostic technologies. For instance, technical performance is related to the anatomic representative images for the diagnostic technologies in radiology. Still, it is not a sufficient source to result in a patient’s change-management by physicians. Moreover, in radiology, much attention is paid to evaluating adverse events (e.g., radiation poisoning, claustrophobia) [6].

In the context of radiology, the HTA could be helpful to adopt new technology, change to old technology, or determine the diagnostic setting in which a technology can be applied.

At IRCCS Ospedale Galeazzi-Sant'Ambrogio, Milan, Italy the EOS System (EOS Imaging, Paris, France) is currently in use, enabling whole-body, weight-bearing, high-quality musculoskeletal radiography exams. The same company has now marketed the EOSedge™, an updated version of the EOS System. Therefore, to evaluate the replacement of the instrumentation in use, an HTA was performed. This paper describes the adoption of HTA process, summarizing a case study on the opportunity to replace the existing diagnostic instrumentation with a novel one.

## Materials and methods

No ethics committee approval was needed for this paper, as no patients are directly involved.

To conduct the HTA, a full HTA model was chosen using the EUnetHTA Core Model^®^ version 3.0 [7]. The HTA Core Model organizes the information into nine domains (Description and technical characteristics of the technology; Health problem and current clinical practice; Safety; Clinical effectiveness; Organizational aspects; Economic evaluation; Impact on the patient; Ethical aspects; and Legal aspects). The information was researched and obtained by consulting the manufacturers' user manuals [8], scientific literature (PubMed, keywords: EOSedge; EOS System, EOS Imaging), and institutional sites (i.e., FDA) for regulatory aspects. Furthermore, the fact that the EOS System was already in use at  IRCCS Ospedale Galeazzi-Sant'Ambrogio has contributed to obtaining helpful information on organizational aspects.

## Results

All nine domains of the EUnetHTA Core Model^®^ helped in conducting the HTA of the EOSedge.

### Description and technical characteristics of the technology

The EOSedge is a digital radiography system that delivers whole-body, high-quality 2D/3D biplanar images covering the complete set of musculoskeletal and orthopedic exams. EOSedge is an updated version of the EOS System (EOS-1st generation). Both instruments are produced by EOS imaging, a manufacturer specialized in 2D/3D orthopedic imaging systems and software solutions for 3D anatomical modeling and surgical planning. In particular, the EOSedge allows for whole-body stereoradiography of the whole skeleton in a weight-bearing position. The main advantages of EOS System are the absence of parallax error and a − 5.6 average lower radiation dose compared to standard cervical spine X-ray examinations [9, 10]. In particular, the Flex Dose™ tool helps reducing radiation dose by 8% compared to the same acquisition not using this application [11]. Moreover, acquisition time of the whole spine is obtained in as little as 4 s with simultaneous frontal and lateral exposure [5].

#### Technical characteristics

EOSedge is built with three main units: the gantry, the acquisition station, and the operator console. The gantry includes an electrical cabinet which contains the system power and communication controls. Two sets of detectors and X-ray tubes are positioned perpendicularly to produce frontal and lateral emission of X-rays, thus generating simultaneously images by scanning the patient over the area of interest. The operator controls the gantry tools from the acquisition station to display images and data. The X-rays emission is triggered manually using the command button integrated into a hand switch on the operator console. This latter command allows the user controlling the system power and X-ray emission during examinations. The images are then stored in a local database and can be transmitted through a digital network for printing and archiving through a standard DICOM protocol.

Radiographers are the professionals who are in charge of operating and correctly performing the EOSedge systems. Patients or their careers do not administer the technology.

EOSedge is designed for a 10-year lifespan.

#### Claimed benefits of the technology

The EOSedge uses automatic exposure control (AEC) which modulates tube current to optimize administration of ionizing radiation dose to patients. The dose reduction principle is possible thanks to the technology applied to scanning method and to detectors. The X-ray beam is highly collimated both at the output from the tube and at the input on the detector. This very precise and sharp collimation significantly reduces the diffused radiation which reaches the detector. Thus, preserving the detection of direct radiation significantly improves the signal-to-noise ratio and allows for a remarkably reduced radiation dose. Collimation is possible both on horizontal and vertical planes. The vertical collimation principle uses green lasers to select the acquisition area. The laser beams are displayed horizontally on the patient to identify the upper and lower limits of the acquisition area. The EOSedge Acquisition application's graphical interface automatically defines the acquisition area. A centering system uses red lasers to check patients’ position in the gantry.

### Health problem and current clinical practice

#### Target population

EOSedge is intended for use in general adult or pediatric radiological exams. The patient should be capable of remaining still for the image acquisition.

#### Clinical management

Patients with spinal deformities and other chronic conditions repeatedly undergo examinations using X-rays during the follow-up of their pathologies [12, 13]. Ionizing radiation exposure increases the risk of developing cancer, especially for younger patients [14, 15]. As an example, young scoliotic female patients may undergo several examinations using X-rays, leading to an increased risk of developing breast cancer [16]. Also, evolutive pathologies such as cerebral palsy or early onset scoliosis also require biplanar X-ray spine exams for evaluation and follow-up due to the high risk of respiratory impairment [17, 18], thus increasing cancer risk and potentially mortality [13].

Clearly, the general tendency is to improve dose reduction strategies in the clinical setting. However, the main standard for spinal deformities follow-up is to perform planar radiography, which includes computed (CR) or digital radiography (DR) [19]. In this setting, it has been reported that the EOS-1st generation allows for organ dose reduction using low-dose and MicroDose protocols as compared to CR and DR [19–23].

#### Comparators in the assessment

EOSedge is an new version of the 1st generation of the EOS System. They have the same intended use, indications, and operation principles [8]. The main difference is that EOSedge uses solid-state photon-counting detectors, compared to gaseous detectors used in EOS 1st generation. The system design has been slightly modified to accommodate the new detectors, but there are no relevant changes to the tube complex and beam-limiting features. Both systems have two sets of detectors and X-ray tubes positioned perpendicularly to acquire simultaneously frontal and lateral radiographs. The two perpendicular acquisition chains consist of high voltage generators, X-ray tubes, collimators, and detectors positioned on two C-shaped arms translating along a vertical axis.

Functional testing reported the equivalent performance of EOSedge compared to the 1st generation EOS System. Bench performance testing was conducted based on the FDA’s Guidance for the Submission of 510(k) for solid-state X-ray imaging devices to verify that EOSedge performs according to specifications and is as safe and effective as the predicate device [25].

Recent research assessed the EOSedge organ radiation dose administration compared to the 1st generation-EOS system focusing on their respective image quality levels [26]. Organ doses were evaluated in an anthropomorphic female adult phantom and a 5-year-old pediatric male phantom using optically stimulated luminescence dosimeters, calibrated in advance within the range of studied energy. Organ doses were recorded on the EOSedge and the Fuji Visionary DRF (Fujifilm Medical Systems U.S.A., Inc., Lexington, MA) [26]. The effective doses resulting from the experiment were compared to the EOS 1st-generation doses reported in literature. Image quality evaluation was performed on end-user images. Quantitative image quality parameters were assessed for all involved modalities on a quality assurance phantom. Qualitative assessment of EOSedge image quality was based on anthropomorphic phantom acquisitions versus the EOS-1st-generation system and clinical images versus the evaluated DR system. For a whole-spine examination performed on the female adult phantom (respectively, the pediatric phantom), an effective dose of 92 microsieverts (μSv) (respectively, 32 μSv) was obtained on EOSedge; these values were compared to effective dose values of 290 μSv (respectively, 200 μSv) from the literature on EOS-1st generation, leading to an effective dose reduction factor of 3-to-6 to EOS-1st generation. EOSedge provides the best compromise between contrast-to-noise ratio (CNR) and dose, with more consistent CNR values than the other tested modalities, in a range of attenuation from 10 to 40 cm of polymethyl methacrylate. The anatomical landmarks which were considered in the follow-up of spinal deformities could be detected in all assessed modalities. Data showed that EOSedge provides significant dose reduction factors for full spine imaging in both adults and children as compared to the EOS System without deterioration of image quality [26].

Table 1 shows the main features of EOSedge and EOS System taken from the literature analysis [26] and user manuals [8], which allow for a comparison between the two technologies (Table 1).

**Table 1:** Comparative features of EOSedge and EOS system

	EOSedge	EOS System
Intended use	General X-ray imaging system	
Indications for use	General radiographic examinations and applications: radiographic acquisition of one or two orthogonal X-ray images for diagnostic purposes in one single scan of the whole body or a reduced area of investigation of a patient in the upright or seated position The MicroDose feature is indicated for imaging with a patient entrance dose of 10 to 90 μGy for assessing global skeletal deformities in follow-up pediatric examinations	
Contraindications	Not designed to perform mammograms, angiograms, or fluoroscopy examinations Not indicated for analysis of spine static in the presence of fusion material or for analysis of the bone–implant interface MicroDose is not indicated for focal skeletal and/or other pediatric abnormalities. MicroDose is not indicated for use in patients with a body mass index over 30	
User population	Trained medical personnel	
Technological characteristics	Digital radiography system in which two sets of detectors and X-ray tubes are positioned orthogonally to generate frontal and lateral images simultaneously by scanning the patient over the area of interest. The two orthogonal acquisition chains consist of HV generators, X-ray tubes, collimators and detectors positioned on C-shaped arms translating along a vertical axis	
Dimensions (l × *w* × *h*)	2.58 m × 2.58 m × 2.706 m (8.5 ft × 8.5 ft × 8.9 ft)	2 m × 2 m × 2.7 m (7.9 ft × 7.9 ft × 10.44 ft)
Weight	2 005 kg (4 420 lb)	1 623 kg (3579 lb)
Accessories	Laser safety barriers Motorized lifting platform Gantry console Laser positioning system Access and stabilization bars Bar code reader	Mechanical platform Laser positioning system Access and stabilization bars
Principles of operation	Tube preheating Daily detector calibration Selection of the patient, the acquisition planes, and the anatomical area, as well as patient positioning Selection of patient morphotype Image preview Display of the exposure area, configuration of the reference planes, and verification and validation of the acquisition parameters Display of unprocessed images and reprocessing with various options Image analysis and export of the exam	
Permanent minimum total filtration (Al equivalent)	1.7 mm Al at 75 kV Additional filtrations: 0.1 mm copper thickness (used for images with or without MicroDose); 0.5 mm copper thickness (used for the Scout View)	1.5 mm Al at 75 kV Additional filtrations: 0.1 mm copper thickness (used for images with or without MicroDose); 1 mm aluminum thickness (used for large patients with LF and power > 28 kW)
Detectors	Direct conversion device – solid-state detector	Direct conversion device – gaseous detector
Pixel depth	17 bits (> 131 000 grey levels)	16 bits (> 65 000 grey levels)
Pixel size	100 μm	254 μm
Resolution	3.7 lp/mm	1.6–1.7 lp/mm
Typical dynamic range	> 100 dB	> 90 dB
Focal spot	Large focal spot: 0.6 × 1.3 at 120 kV – 100 mA	Large focal spot: 0.6 × 1.3 at 120 kV -100 mA Small focal spot: 0.4 × 0.7 at 120 kV—50 mA
Linear scanning speed (cm/s)	From 4.1 to 32.5	From 3.8 to 30.5
Average acquisition time	8 s for the whole spine and 15 s for the entire body	5 to 10 s for the whole spine and 20 s for the whole body
Organ dose deposition	92 μSv in female adult phantom 32 μSv in pediatric phantom	290 μSv in female adult phantom 200 μSv in pediatric phantom

#### The scale of current use of the technology

Best practice guidelines for bracing adolescent idiopathic scoliosis (AIS), which were recently published, support the use of EOS examinations for such patients [27]. Pediatric and adult patients with spinal deformities undergo to diagnostic examinations exposing them repeatedly to ionizing radiations. It is proven that ionizing radiation can increase risk of cancer, particularly in younger subjects who have rapidly dividing cells that may be more susceptible to DNA damage. CT administers 10 to 100 times higher radiation dose than conventional radiographs. The use of CT imaging is growing constantly in the United States, representing about 50% of overall medical ionizing radiation exposure. Early onset scoliosis patients are at risk of high cumulative ionizing radiation exposure: This is related to their young age at diagnosis and possible coexistence of multiorgan system involvement which happens in neuromuscular, congenital, or syndromic patients. Biplanar X-ray examinations reduce ionizing radiation exposure, and overall levels of exposure from radiographic imaging are low compared to conventional radiographs and even lower compared to CT imaging.

#### Growing adoption of the technology

EOSedge has gained significant trust by healthcare providers, as 6 out of 10 equipment orders were placed for this system rather than for Eos System [28]. Since its launch in December 2019, EOSedge has been very well received by the medical community, promising future solid opportunities [28]. In North America, installations returned nationwide in academic centers, local hospitals, and orthopedic private practices [28]. EOS imaging continued its expansion in Europe, despite the COVID-19 pandemic: The new EOSedge system was installed in key reference sites in France (Bordeaux, Ajaccio) and Germany (Hamburg) within the renowned Asklepios health network [28]. EOS imaging maintained momentum in the Asia–Pacific region by enlarging its installed base in India, Korea and Singapore while taking new orders, including the flagship product, EOSedge, in Australia [28].

This trend is expected to progressively grow as thirty-eight top orthopedic surgeons, physiatrists, orthotists, physical therapists, and research scientists from seven countries recently published a consensus paper in the Spine Deformity Journal, in which they recommend the use of low-dose biplanar radiography to follow-up AIS patients treated with spinal braces [27].

### Safety

At baseline, 30% of adults are expected to develop cancer in their lifetime. It is unclear whether childhood radiation exposure from diagnostic imaging leads to increased tumor risk [29] as cancer has multifactorial origin (inherited genetics, environmental exposure, obesity, and alcohol use). Therefore, it is difficult to determine whether patients develop cancer because of medical radiation exposure or other factors. Even though association between radiation exposure and cancer is proven, stating causality is more challenging. In contrast to adults, children are thought to be at increased risk of detrimental effects of radiation, as their cells are more rapidly replicating [30].

Of all human radiation exposure in the United States, approximately 50% is due to medical imaging [31]. Given that, physicians should make any efforts reducing patients and healthcare professionals to radiations.

EOSedge uses automatic exposure control with tube current modulation to optimize dose administration in patients, thus providing low levels of radiations (i.e., far below 100 μSv); therefore, it is not thought to cause any specific undesirable effects and side effects under normal use conditions. If the examination involving ionizing radiations is medically indicated, the risk to children’s of not doing the procedure is greater than the risk of potential harm. Weekly natural background radiation is estimated at around 46 μSv [32]; considering the estimated effective dose, EOSedge examinations are equivalent to 5 days of natural radiation for children and two weeks for adults [26].

EOSedge is manufactured following the safety standards in force (Table 2); nevertheless, X-rays are harmful when unqualified and untrained technicians use the equipment. As a result, every precaution must be taken to prevent unauthorized or unqualified persons from using this device to prevent them from endangering themselves and others.

### Clinical effectiveness

EOSedge is intended for general radiology examinations, particularly of the skeleton, except for evaluating pulmonary nodules, fluoroscopy examinations, angiograms and mammograms. As it generates a full body scan and constructs a three-dimensional model from synchronously acquired lateral and posteroanterior images, it has been validated for scoliosis, sagittal balance, pelvic and lower-limb deformity and pathology in adult and pediatric populations due to the reduction of radiation exposure for patients who require repeated radiological examinations over time [8, 9].

In addition to the decreased radiation exposure [33, 34], its advantages include developing 3D reconstructions and 3D rotational analysis [35]; the image quality, compared with DR and CR techniques, is enhanced, particularly on the lateral view. Evaluating spinal deformities in the transverse plane can provide valuable information on the severity of scoliosis and impacts therapeutic decisions [36]. A recent study investigated the long-term health-related quality of life (HRQoL) in patients with idiopathic scoliosis, showing a significantly decreased HRQoL and work ability in patients with idiopathic scoliosis 40 years after first diagnosis [37]. Thus, the possibility of performing serial radiographs to confirm the initial diagnosis and to follow up curve progression over time with very low radiation exposure remains an essential objective for all medical professionals [36]. The main disadvantage, albeit minimal, of biplanar slot scan imaging is the risk of motion artifacts, as patients must remain still for a slightly longer period compared to conventional radiography [38].

### Organizational aspects

#### Investments, disinvestments, and changes in service organization

The investments needed are related to acquiring the EOSedge and divesting the EOS System already used at IRCCS Ospedale Galeazzi-Sant'Ambrogio. Apart from personnel training, who will use the new equipment, there is no requirement for any further investment in infrastructure before the new technology can be installed.

Concerning the replacement timing, to better meet customer expectations and improve its working capital, EOS imaging changed its commercial cycle at the beginning of 2019 by organizing the delivery of EOS Systems during the installation phase and no longer just after receiving the equipment order [28]. Installations usually occur 3–12 months after the order; thus, a similar delivery delay occurs.

From an organizational point of view, replacing the existing procedure relating to patient management is unnecessary; there may be a variation in the volume of exams that can be done, as system is faster.

### Economic evaluation

An essential difference between the assessed radiography system is that, while the EOSedge is a newly released tool, the EOS System in use is at risk of obsolescence. In the specific context, these last considerations critically impact the cost (with a difference of about 75% between the quotation of the two systems).

To our knowledge, no cost-effectiveness analysis has been carried out about EOSedge. Instead, the cost-effectiveness analyses on the EOS System are available, concluding that the technique might not be considered a cost-effective intervention [39, 40]. In particular, Faria R et al. evaluated the loss of quality-adjusted life years (QALY) due to cancer attributable to radiation exposure that patients underwent due to a diagnosis or long-term monitoring, comparing standard X-ray [40], concluding that EOS imaging was not cost-effective in terms of QALYs.

### Impact on the patient

As with many other diagnostic tests, patient does not perceive technology value directly. For this reason, we conducted a brief and empirical interview to four orthopedists and two physiatrists who are currently involved in the use of the EOS System at IRCCS Ospedale Galeazzi Sant'Ambrogio, asking the following questions:

1. Do you think EOS is better than a normal spine teleradiography? If yes, why?
2. When you request EOS in place of teleradiography, do you explain to the patient or parents why? What words do you use?
3. Do you think the patient or parents perceive the difference and/or added value of EOS?
4. Approximately what percentage of patients to whom you have recommended it actually perform EOS instead of an X-ray?

They in general have a very positive opinion of the EOS system, with their answer being herebelow summarized:

1. EOS is better than standard digital radiography for four basic reasons: the reduced exposure to ionizing radiation; the absence of the “distortion” at the extremes of teleradiography induced by the single source compared to EOS acquisition always orthogonal to the subject; and thanks to this, the possibility of 3D spatial reconstruction of the spine, which allows more detailed studies of deformity for scientific purposes and of global posture with automatic analysis of pelvic parameters for clinical purposes; in parallel with this, the greater reliability of patient positioning and thus the possibility of clinical comparison for analysis of groups of patients.
2. We always explain the reason, stating that in the follow-up of a spine deformity there is a need for repeated radiographic checks, and therefore the use of EOS allows on the one hand to reduce the overall exposure at the end of treatment and on the other hand to allow even closer follow-ups if necessary.
3. We are totally convinced patients or their parents perceive the importance of EOS, at the point they are available to travel from distant cities to perform it.
4. In about 90% of cases, they want to perform the requested exams with EOS System rather than with a conventional radiography system.

### Ethical aspects

As in many aspects of medicine, medical interventions are not always free from side effects; the risks and benefits are often analyzed. In the case of radiological examinations, there are risks associated with using X-ray imaging, which uses ionizing radiation to generate images of the body. Risks from exposure to ionizing radiation include a slight increase in the possibility of developing cancer and tissue effects (i.e., cataracts, skin reddening, and hair loss) at high levels of radiation exposure. Moreover, analyzing the risk–benefit ratio, it is considered that if the diagnostic radiology examination is medically indicated, the risk to the children of not doing the procedure is greater than the risk of potential harm.

Therefore, reducing unnecessary radiation exposure should be a priority without compromising the quality of care, according to the *As Low As Reasonably Achievable* (ALARA) principle [41]. The EOSedge MicroDose protocol significantly reduced the delivered dose while maintaining interpretable image quality and good interrater agreement of 3D spine measurements.

### Legal aspect

EOSedge is designed and certified to conform to IEC 60601-1 and collateral standards. Software verification and validation testing were also conducted [8]. Table 2 shows the international standards to which EOSedge complies regarding the safety of electromedical devices. Moreover, EOSedge is equipped with 635 nm red-colored lasers of class 1, 1520 nm green-colored lasers of class 1, and 905 nm laser security curtains of class 1, following the standard IEC 60825-1: 2014 and 21 CFR1040-10 [8].

**Table 2:** Radiation dose comparison between EOSedge and EOS system

IEC 60601-1	Medical electrical equipment—Part 1: general requirements for basic safety and essential performance
IEC 60601-1-2	Medical electrical equipment—Part 1–2: general requirements for basic safety and essential performance—Collateral standard: Electromagnetic disturbances—Requirements and tests
IEC 60601-1-3	Medical electrical equipment—Part 1–3: general requirements for basic safety and essential performance—Collateral standard: radiation protection in diagnostic X-ray devices
IEC 60601-1-6	Medical electrical equipment—Part 1–6: general requirements for basic safety and essential performance—Collateral standard: suitability for use
IEC 60601-2-28	Medical electrical equipment—Part 2–28: special requirements for basic safety and essential performance of tube assemblies equipped for medical diagnosis
IEC 60601-2-54	Medical electrical equipment—Part 2–54: special requirements for the basic safety and essential performance of X-ray equipment used for radiography and fluoroscopy
IEC 60825-1 and 21 CFR1040-10	Compliance of lasers

The software, acquired under license, is protected by law on industrial and intellectual property in its country of origin, following French and European legislation and applying international agreements.

## Discussion

Health technology assessment (HTA) leads to well-rounded decisions on the adoption of new technologies, covering a broad range of assessment domains, including medical, social, economic, and ethical aspects. In particular, the EUnetHTA Core Model^®^ offers several advantages that make it a valuable tool to conduct HTA. One of the main strengths of the EUnetHTA Core Model^®^ is its ability to standardize the HTA across different European countries, making the assessments reproducible and understandable, increasing trust among stakeholders, including policymakers, healthcare providers, and patients. It facilitates the sharing of information and best practices, which can lead to more efficient use of resources and improved capacity building in HTA practices.

Despite its structured approach, the EUnetHTA Core Model^®^ is designed to be adaptable to various types of health technologies, including medical devices, pharmaceuticals, and procedures, as the model places significant emphasis on patient and clinical outcomes.

The use of the EUnetHTA Core Model® for the assessment of the EOS system made it possible to highlight that the EOS technologies may be a viable alternative to conventional radiographs [6, 36, 42]. EOSedge has the same intended use and similar indications for use, technological features, and principles of operation as the EOS System (Table 1). EOSedge provides significant dose reduction factors for whole spine imaging compared to the EOS System without compromising image quality [26].

The patient’s direct benefits are the relatively low dose of radiation, resulting in a decrease in undesirable effects and side effects under normal conditions of use. However, it is essential to point out that the literature on radiation dose can be difficult to interpret because of the various quantities and units of radiation measurement used [13]. CT dose index (CTDI) and dose length product (DLP) may be listed on radiology reports but are not easily translatable into the patient’s absorbed dose [43]. CTDI does account for both the dose delivered by the radiograph beam as well as scatter from surrounding irradiated tissues. Milligray measures the amount of radiation absorbed by a material, such as an organ or body part, while Millisieverts measure the effective dose estimations, which helps compare different sources of exposure (i.e., radiological instrumentation, natural background exposure). Moreover, the organ/tissue doses are estimated and multiplied by a weighting factor to calculate the effective dose, which considers organ/tissue radiosensitivity.

Regarding the impact of EOS imaging on patient outcomes, the literature review shows that most studies aim to establish technical ability without evaluating their ability to improve patient outcomes [39, 40, 44]. Therefore, it would be necessary to conduct clinical studies investigating the impact of EOSedge on patient outcomes.

### Limitations and alternatives of the EUnetHTA core model^®^

The EUnetHTA Core Model^®^ is comprehensive but can be resource-intensive, requiring significant time and expertise to fully implement. This complexity may limit its use in resource-limited environments. The INAHTA checklist [45], developed by the International Network of Agencies for Health Technology Assessment (INAHTA), provides a more direct and less resource-intensive approach than the EUnetHTA Core Model^®^. It is designed to be adaptable and is primarily used to facilitate health technology assessments by smaller or resource-limited organizations. Another limitation could be the integration of the results of an EUnetHTA-based evaluation with local health policies and practices, particularly in regions with different health priorities or regulatory contexts. In fact, several Asian countries have developed the HTAsiaLink model to promote effective and efficient health technology assessment practices [46]. This model is best suited to healthcare settings in Asia. It focuses on regional collaboration and capacity building, offering a more culturally and economically appropriate framework for those regions.

## Conclusion

Implementing the culture and diffusion of HTA in radiology favors the reduction of the gap between technological innovation and decision-makers. Significantly, the HTA, being adapted to the specific context, can support the choice of health technologies of decision makers at the micro (clinical managers), meso (managerial managers) and macro (policymakers) levels. HTA is also essential in radiology because the instruments are used for several years; therefore, the selection should not be based on only the technical ability and the costs, but a global approach is needed to allocate the resources efficiently.

Finally, to be more effective, an HTA of diagnostic technologies in radiology must consider the risk of exposure and factors of biological variability, as in the estimation of the organ/tissue doses are also implicated in weighting factor, which considers organ/tissue radiosensitivity. Thus, integrating a sex and gender analysis into HTA would promote equity in healthcare and enhance the validity and applicability of HTA [44].

## Abbrevations

HTA: Health technology assessment
CNR: Contrast-to-noise ratio
CR: Computed radiography
DR: Digital radiography
μSv: Millisieverts
µGy: Milligray
QALY: Quality-adjusted life years
ALARA: As low as reasonably achievable
